# Repertory of the Back

**Published:** 1899-12

**Authors:** E. H. Wilsey

**Affiliations:** Parkersburg, W. Va.


### REPERTORY OF THE BACK.

By Dr. E. H. Wilsey, Parkersburg, W. Va.

### KIDNEY REGION.

**Abdomen,** pain in region of kidney, extending downward and toward abdomen, aggravated at night, Tellurium.

— sudden pain in region of right kidney, extending to epigastric region, then spreading over abdomen, causing warmth in abdomen, Hydroc-ac.

**Above**, sticking above region of right kidney, Lyco.

**Aching** across back and in kidneys, Med.

— in kidney region and in lower part of left loin when walking, Apoc.

— on taking a drive in kidney region and in loins, Calc-c.

— and drawing pain from kidney to sacrum and in thighs and legs; better by drawing the legs up, Cinnab.

— pain in right kidney and stomach, *Crotalus.*

— dull aching pain in sacrum, running up into kidney, Eup-pur.

— in kidney at 10 P. M., Helon.

— in kidney region at 4 P. M. while walking, Hyperi.

— in region of left kidney as if urine had been retained too long; worse by sitting, better by pressure, but worse afterward, Pallad.

— frequent urging to urinate and aching in bladder, and next day aching in left kidney when sitting, Pallad.

— heavy aching across kidneys mornings on waking, Æthusa., Ham.

**Across,** drawing across region of kidney, with warmth during the fever, Lach.

— from 4 A. M. till evening pain in both kidneys and across hips; severe and a little burning; better at 9 P. M., Lyssin.

— and in kidneys, aching, Med.

— pain across kindneys, then diuresis, Morphinum.

— pain across region of kidneys, Still.

— heavy pain across kidneys in morning, urine high-colored and not as easily voided as usual, Sang.

KIDNEYS.

**Acute** pain in region of kidneys, Codein.

**Afternoon,** pain in right kidney in afternoon, Bad.

**Arms,** sharp pain in region of kidneys when moving arms, Ant-t.

**Awake,** pain in kidney keeping him awake at night, *Can-ind.*

**Before** urinating, a pain in kidney region, Tabacum.

**Bladder,** pain in ureters extending to bladder, *Canth.*

— stitches from kidney into bladder, *Berb.*

— sticking from kidney along ureter to bladder, Cocc-c.

— drawing pain in right kidney extending along ureter to bladder, waking him at 5 A. M., Cocc-c.

— soreness of kidney region gradually extending to bladder, with frequent urging to urinate, with increased dark urine, Cocc-c.

**Burning** pain in kidney, with profuse urine, smarting and burning in urethra, then profuse micturation of clear urine and along ureters, as if boiling water was poured through them, Cedron.

— in region of left kidney, deep, extending toward right side, Lachn.

— in kidneys, *Can-ind.*

— 4 A. M. till evening pain in both kidneys and across hips, severe and a little burning, better at 9 P. M., Lyssin.

— pain in left kidney, with burning in all urinary organs, Euphorb.

— drawing pain in region of kidneys, *Tereb.*

— in renal region with frequent burning and micturation, increased and drawing along ureters, Thuja.

**Breathing,** stitches in region of kidneys arresting breathing in P. M., Croton.

**Breath,** heat in kidney region on taking a long breath, Sepia.

**Bruised** feeling in kidney region, Physos.

— pain in kidneys and above kidneys, extending to gluteal and thighs, worse by motion and touch, especially stooping, Clem.

— pain in lumbar region and across kidneys from 2 P. M. till 4 P. M., Conval.

### KIDNEYS.

**Chills,** creeping in region of right kidney, throbbing, contracting, drawing, and relaxing, as if caused by icy-cold insects with claws, Med.

**Constriction** spasmodic from kidney to bladder, Nit-ac.

**Creeping** sensation occasionally in region of left kidney, Med.

**Cramp-like** pain in hip from kidney to thighs, with sensation as if hip joint was fastened by iron clamps, *Colocy.*

— spasmodic pain in region of kidneys streaming out from both sides in afternoon, when sitting, to be compared with cramp-like pain after an injury to testicles by an emission of flatus, Cocc-c.

**Cramp,** shooting pains from right kidney down to knee like cramp, *Ip.*

**Crawling,** heavy pain in kidney region, with darting pain occasionally, with crawling sensation, Dirca-p.

**Cutting** at times in right kidney region, at times a drawing pressure, Zinc.

— violent pain in region of kidneys when lifting arm or blowing nose; pain in back jerking, cutting, and shooting, *Calc-ph.*

— above region of left kidney, Merc.

— and dragging down from kidneys before urinating, Graph.

**Darting** pain occasionally through kidney, Cund., Dirca-p.

— pain in region of left kidney, darting over left hip, especially when spine is pressed upon, Med.

— pain in left kidney on waking at 6 A. M., then in right, with heavy pain in left testicle, Stilling.

**Deep,** penetrating, dull stitches in region of right kidney, worse during inhalation, Cyclamen.

**Digging** in kidney region, evening in bed, then sticking, making him jump, Cocc-c.

**Drawing** in region of kidney, extending along loins when sitting, Ruta.

— in right kidney, extending to right hip, Tereb.

— burning in renal region, with frequent burning micturition, increased and drawing along ureters, Thuja.

— across region of kidney, with warmth during the fever, Lach.

KIDNEYS.

**Drawing,** creeping chills in region of right kidney, throbbing, contracting, drawing, and relaxing, as if caused by icy cold insects with claws, Medor.

— and aching pain from kidneys to sacrum, and in thighs and legs, better by drawing up legs, Cinnab.

— pain in right kidney, extending along ureter to bladder, waking at 5 A. M., Cocc-c.

— in region of right kidney, with pressure, Iod.

**Drive**, aching in kidney region on taking a drive, also in loins, Calc-c.

**Formication** in kidneys, Hydrocotyle.

**Fluttering** sensation in kidney region, sometimes one and then the other, without pain or uneasiness, *Chimaphila-umb.*

**Griping** in region of kidneys, Cadmium.

**Heat** in kidney region on taking a long breath, Sepia.

— and tension in renal region, even while sitting; walking tires him, Nat-m.

**Heavy** pain in kidney on waking, Oxytropis.

— pain across kidney in morning, urine high colored and not as easily voided as usual, Sang.

— darting pain in left kidney on waking at 6 A. M., then in right with heavy pain in left testicle, Stilling.

— pain in kidney region with crawling sensation and occasional darting pain, Dirca-p.

— pain over right kidney, preventing sleep first part of night, Equise.

**Heaviness,** obstruction in right kidney with heaviness, Hydrocotyle.

**Hot**, sensation as if kidneys were two bags of hot water, Helon.

**Itching** stitches in kidney region, Staph.

**Jerking** under left kidney, Astacus.

**Laughing**, pain in kidneys when laughing, *Can-ind.*

**Left**, pain in region of left kidney, Elater.

**Morning,** heavy aching in morning across kidneys on waking, Æthu.

**Movements** in region of kidneys, Camph.

KIDNEYS.

**Pain** in region of left kidney, Cepa., Chloro., Clem., Zinc.
— in right kidney at 9 P. M., then in left, extending down to side of sacrum, Equisetum.
— in left kidney with burning in all urinary organs, Euphorb.
— desire to pass water with pain in kidney region, urine albuminous, feet and legs dropsical, *Fer.*
— in kidneys, Gnaphal.
— in region of kidneys, Bovis., Papaya-vul., Kali-brom.
— sudden pain in region of right kidney. extending to epigastric region, then spreading over abdomen, causing warmth in abdomen, Hydroc-ac.
— in right kidney, Iris-v., Stilling, Lyco.
— in kidneys with tenderness, Oxytropis.
— in region of left kidney, running around ilium, and bruised feeling in kidney region, Physos.
— great pain in back and region of kidney, aggravated after urinating, Syph.
— in region of kidney before urinating, Tabacum.
— burning, drawing pain in region of kidney, *Tereb.*
— in region of kidneys, extending downward to abdomen, worse evenings and night, Tell.
— in right kidney from 4 to 5 P. M., Lyssin.
— in kidney region with profuse urination, Nat-ars.
— in kidney during apyrexia, *Nat-m.*
— in kidney region, as if ulcerated, Can-Sat.
— drawing pain in kidney region extending to inguinal glands with anxious nausea at pit of stomach, *Can-Sat.*
— in kidney and abdomen with such pain on urinating that he could not pass a single drop without moaning and screaming, *Canth.*
— in kidney region late in evening, *Canth.*
— in kidneys with profuse urine, smarting and burning in urethra, then profuse micturition of clear urine and burning along ureters as if boiling water were poured through them, Cedron.
— in kidney region with urging to urinate, Ars-h.

### KIDNEYS.

**Pain,** oppressive pain in kidney region, Cocc-c.

**Pinching** in kidney region, Caustic., Zinc.

**Pressing** in region of kidneys, Lyssin.

— soreness in region of right kidney when pressing back against carriage seat, Amm-benz.

**Pressure** in region of right kidney, Mar-v.

— in region of right kidney with drawing, Iod.

— or stitches in region of both kidneys, Kali-c.

— or sticking in kidney region, worse by pressure, *Lyco.*

— or severe pain in back in kidney region, better by pressure across back with arms crossed, Viburnum-op.

— in kidney region, worse turning body with sticking, Berb.

— in region of kidneys painful to pressure, *Cocc-c.*

**Pulling** sensation, a feeling over right kidney as if something pressed hard against it; better by pressure, but leaves a pulling sensation, Amm-brom.

**Pulsating** stitches in region of loins and kidneys, Sul.

**Rectum**, sticking in kidney region extending into rectum, Lyco.

— pain in kidney penetrating into right iliac fossa, then shooting through to sigmoid flexure of colon, then extending to rectum, Sang.

**Rise,** when she stoops cannot rise again without violent pain in kidney region, Med.

**Rising,** backache in kidney region when rising in morning, Ham.

**Sacrum,** dull aching pain in sacrum running up into kidney, Eup-pur.

**Sensation** of pain in back and kidney and lower portion of spine, starting from lumbar vertebræ, Fer-i.

**Severe** pain in back in kidney region better by pressing arms across back, Vib-op.

**Sharp** pains in kidney region when moving arms, Ant-t.

— pains in region of right kidney leaving soreness, Convall.

— pain in kidney region as if nail had been driven in side vertebræ, Cinnab.

— pain in region of left kidney (plunging), Myrica.

KIDNEYS.

**Shooting** sharp pains, first left then right renal region extending down thighs, worse on motion, *Kali-b.*

— pains from right kidney down to knee like cramp, *Ipec.*

— pain in kidney region penetrating into right iliac fossa, then shooting through to sigmoid flexure of colon, then extending to rectum, Sang.

**Shot,** sudden aching in kidney region at night when undressing, as if, Helon.

**Sitting,** tension in kidney region even when sitting, with heat, and walking soon fatigues, Nat-m.

— frequent urging to urinate and aching in bladder, and next day aching in left kidney when sitting, Pallad.

— sticking in region of left kidney when sitting, Dig.

— aching in region of left kidney as if urine had been retained too long, worse on sitting, better from pressure, but worse afterward, Pallad.

— stitches in kidney region when sitting down, Valer.

**Sleep,** heavy pain over right kidney, preventing sleep in forepart of night, Equisetum.

**Sleeplessness,** stitching in region of left kidney, waking him at four A. M., then tossing about and sleeplessness, Cinnab.

**Soreness** of kidney aggravated in right, Equisetum.

**Sore** pain in small of back in kidney region all day, Hydrast.

— spot over right kidney, Amm-brom.

**Soreness** slight in region of kidneys when bringing muscles into action, Apoci-can.

— soreness in region of kidneys, Mancin.

— of kidney region gradually extending to bladder, aggravated into sphincter with frequent urging to urinate, with increased high urine, Cocc-c.

— weakness, pain and soreness in kidney region, Phyto.

**Spasmodic** constriction from kidneys to bladder, Nit-ac.

— pain in region of kidneys streaming out from both sides in afternoon when sitting, to be compared to the cramp-like pain after an injury to testicles, better by emission of flatus, Cocc-c.

KIDNEYS.

**Squeezing** in left kidney region, Hyperi.
**Sticking** in left renal region, Croton.
— in region of kidneys, Dig., Vipera.
— in kidneys and liver, Sepia.
— inward in kidney region, Stann.
— in region of right kidney, Lob-i.
— in region of left kidney, Lyco.
— above region of right kidney, Lyco.
— in kidney region, worse by pressure, *Lyco.*
— from kidneys along ureters to bladder, Cocc-c.
— and pricking pain in region of kidneys, Carbo-an.
— pressure in kidney region, worse turning, with sticking, Berb.
**Stiffness,** dull pains in region of kidneys and stiffness in loins, Benz-a.
**Stinging,** sharp stinging pains in kidney region, *Kali-b.*
**Stitches** in region of kidneys, Ran-scl.
— itching stitches in region of kidneys, Staph.
— pulsating stitches in region of loins and kidneys, Sul.
— in kidney region when sitting, Valeriana.
— in kidney region, Zinc.
— in renal region, Phos-ac.
— from kidneys into bladder, *Berb.*
— in region of kidney arresting breathing, Croton (in P. M.).
— in left kidney, Gambog.
— pressure or stitches in region of both kidneys, Kali-c.
— in right kidney region, Canth.
**Stomach**, aching pains in right kidney and stomach, *Crotalus.*
**Stoops,** when she stoops cannot rise again without violent pain in region of kidney, Medor.
**Stooping**, tearing in region of kidney, worse by stooping, Raphanus.
**Stretching**, tired sensation in region of kidneys, desire to rub parts, with much stretching, *Mancin.*
**Stooping**, bruised pain in and above kidneys extending to gluteal region and thighs, worse by motion and touch, especially stooping, Clem.

### KIDNEYS.

**Tearing** in region of right kidney, worse by stooping, Raphanus.
— from both sides of vertebræ into kidneys, worse from motion of trunk, Stann.
— in region of right kidney, *Lyco.*
**Tenderness**, pain in kidneys with tenderness, worse in right, Oxytropis.
— weakness and tenderness of kidneys on pressure, Plant.
**Tension** in kidney region when sitting with heat, and walking soon fatigues, Nat-m.
— and pain in region of kidneys, *Colch.*
**Testicle**, darting pain in left kidney on waking at 6 A. M., then in right with heavy pain in left testicle, Stilling.
**Throbbing**, twitching in right kidney region with throbbing, Canth.
— and thumping in region of right supra-renal capsule, seeming to come from an abscess or sore spot just below fifth rib, right side under breast, Med.
**Through**, darting pain occasionally through kidneys, Cund.
**Tired** sensation in region of kidney, desire to rub parts, much stretching, *Mancin.*
**Toothache**, pain in loins like toothache when she stands, which makes her feel sick and good for nothing, *Colchi.*
**Twitching** in right kidney region with throbbing, Canth.
**Undressing**, sudden aching at night on undressing as if shot, Helonias.
**Uneasiness** and weight in kidney region, *Helonias.*
**Urgent**, dull pain in region of right kidney with urgent desire to urinate, *Equisetum.*
**Urinating**, great pain in region of kidney, worse by urinating, Syph.
**Urination**, pain in kidney region with profuse urination, Nat-ars.
**Ureters**, pain in ureters extending into bladder, *Canth.*
**Violent** pain in region of right kidney, could lie only on his back, worse from pressure and motion, *Colchi.*
**Waking**, heavy pain in kidney on waking, Oxytropis.

KIDNEYS.

**Waking,** darting pain in left kidney on waking at 6 A. M., then in right kidney with heavy pain in left testicle, Stilling.

**Walking,** aching in kidney region and in lower part of left loins when walking, Apoc-can.

— at four P. M., aching in renal region while walking, Hyper.

**Water,** sensation as if kidneys were two bags of hot water, Helonias.

**Weakness,** pain and soreness in region of kidney, Phyto.

— in region of kidney, loins, and sacrum, Lyss.

— in left kidney region, Cund.

